# Vascular endothelial growth factor in children with neuroblastoma: a retrospective analysis

**DOI:** 10.1186/1756-9966-28-143

**Published:** 2009-11-06

**Authors:** Gordana Jakovljević, Srđana Čulić, Jasminka Stepan, Aleksandra Bonevski, Sven Seiwerth

**Affiliations:** 1 Department of Hematology and Oncology, Pediatric Clinic, Children's Clinical Hospital Zagreb, Zagreb, Croatia; 2 Department of Pediatric Hematology, Oncology, Immunology and Medical Genetics, Pediatric, Clinic, Clinical Hospital Center Split, Medical School University of Split, Split, Croatia; 3 Institute of Pathology, Zagreb University School of Medicine, Zagreb, Croatia

## Abstract

**Background:**

Despite aggressive therapy, advanced stage neuroblastoma patients have poor survival rates. Although angiogenesis correlates with advanced tumour stage and plays an important role in determining the tumour response to treatment in general, clinical data are still insufficient, and more clinical evaluations are needed to draw conclusions. The aim of this study was to evaluate vascular endothelial growth factor (VEGF) expression in patients with neuroblastoma, determine whether it correlates with other prognostic factors and/or therapeutic response, and to assess should VEGF be considered in a routine diagnostic workup.

**Materials and methods:**

VEGF expression was determined by immunohistochemistry using anti-VEGF antibody in paraffin embedded primary tumour tissue from 56 neuroblastoma patients. Semiquantitative expression of VEGF was estimated and compared with gender, age, histology, disease stage, therapy, and survival. Statistical analyses, including multivariate analysis, were performed.

**Results:**

VEGF expression correlated with disease stage and survival in neuroblastoma patients. Combination of VEGF expression and disease stage as a single prognostic value for survival (P-value = 0.0034; odds ratio (OR) (95%CI) = 26.17 (2.97-230.27) exhibited greater correlation with survival than individually. Hematopoietic stem cell transplantation significantly improved survival of the advanced stage patients with high VEGF expression.

**Conclusion:**

VEGF expression should be considered in a routine diagnostic workup of children with neuroblastoma, especially in those more than 18 months old and with advanced disease stage. High VEGF expression at the time of disease diagnosis is a bad risk prognostic factor, and can be used to characterize subsets of patients with an unfavourable outcome.

## Background

Neuroblastoma (NB), a paediatric solid tumour of neural crest origin, is the most frequent extracranial solid malignancy in children. Despite intensive multimodal therapy, the prognosis of patients older than 1 year with advanced disease remains poor, with long term survival less than 40%. A consensus was reached in determining the neuroblastoma risk stratification schema considering age, stage and N-*myc *status [1]. In general, angiogenesis plays an important role in the progression and metastasis of malignant tumours [2]. In neuroblastoma, tumour vascularity is correlated with an aggressive phenotype [3,4]. Pro-angiogenic factors are differentially expressed in high-risk neuroblastoma [5,6]. Vascular endothelial growth factor (VEGF) is a specific endothelial cell mitogen that stimulates angiogenesis and plays a crucial role in tumour growth [7]. Overexpression of VEGF has been demonstrated in neuroblastoma, nephroblastoma, as well as in other cancers, such as colon, breast, brain, lung, malignant pleural mesothelioma, esophageal and gastric carcinomas [8-10]. In adult solid tumours VEGF expression has been successfully evaluated by immunohistochemistry, and has been reported to be an independent prognostic factor [11-15]. Recent studies have validated inhibition of VEGF as an effective antiangiogenic therapy in some of these cancers [16-18]. Although several preliminary studies have demonstrated that expression of angiogenic growth factors, including VEGF, correlate with a high-risk phenotype in neuroblastoma, clinical data are still insufficient to draw conclusions [5,9,19-21]. Therefore, further clinical studies, are needed to evaluate the possible significance of these factors for use in a routine clinical practice. Preclinical studies also suggest that antiangiogenic strategies may be effective in the treatment of neuroblastoma [22,23]. Whether inhibition of angiogenesis is a realistic approach for preventing dissemination of neuroblastoma, remains to be determined. In addition, phase I clinical trials (COG study) using the human anti-VEGF antibody, bevacizumab, in pediatric patients with refractory solid tumours reported promising results [24].

The aim of this study was to evaluate VEGF expression in patients with neuroblastoma and determine whether it correlates with other prognostic factors and/or therapeutic response. Also, we tried to assess should VEGF be considered in a routine diagnostic workup of children with neuroblastoma. Maybe these results could help in the planning further follow-up strategies and antiangiogenic therapy trials.

## Materials and methods

### Patients and tumour samples

Neuroblastoma tissue samples (n = 56) included in this study were retrieved from the archives of the Institute of Pathology Medical School University of Zagreb, Croatia. They were obtained from patients treated at the Children's Clinical Hospital Zagreb between 1995 and 2008 at the beginning of disease (first biopsy). Clinical staging was classified according to The International Neuroblastoma Staging System (INSS) [1,25]. Histopathological grading was classified according to Shimada System and Shimada Age-based Pathologic Classification [26,27]. All the histological samples underwent a revaluation and new grading (SS). Patients with stage 1, 2 and stage 4s disease (19 patients) were treated with surgery alone, or surgery and moderate-dose chemotherapy. Patients with stage 3 and 4 (37 patients) were treated with surgery combined with intensive, multiagent chemotherapy either with or without radiotherapy and/or metaiodobenzylguanidine (MIBG) therapy. Fourteen patients with advanced disease, and 3 patients with localized disease with *N-myc *amplification tumour received megatherapy (myeloablative chemotherapy) followed by autologous or allogeneic hematopoietic stem cell transplantation. As hematopoietic stem cell transplantation for our high-risk patients was started in 1999, there were 2 groups of high risk patients, either treated with or without stem cell transplantation (Table 1).

**Table 1:** Patient characteristics

Characteristics	No. patients
**Total number**	**56**
	
Gender	
Male	35
Female	21
	
Age	
Median 35.5 months	
Range 2 months - 12 years	
>18 months old	36
≤ 18 months old	20
	
Histologic subtype	
Stroma-rich	
Well differentiated	3
Intermixed	10
Focal nodular	3
Stroma-poor	
Undifferentiated	30
Differentiating	10
	
Histology	
Favourable	23
Unfavourable	33
	
Stage	
1	3
2	15
3	20
4	17
4s *	1
	
Treatment	
S	3
S/CTH	32
S/CTH/MIBGT	2
	
S/CTH/RT	2
S/CTH/BMT	14
S/CTH/MIBGT/BMT	2
S/CTH/BMT/RT	1
	
Survival	
Alive	35
Dead	21

Abbreviations: 4s * in infants with small primary tumours and metastatic disease involving the skin, liver, limited infiltration of the bone marrow, and can spontaneously regress; S, surgery; CTH, chemotherapy; MIBGT, metaiodobenzylguanidine therapy; BMT, bone marrow transplant; RT, radiotherapy

### Immunohistochemistry

Immunohistochemical analysis was performed on formalin-fixed paraffin-embedded tumour sections. Following surgery, specimens were fixed in 4% buffered formalin for at least 24 h, embedded in paraffin, and stored at room temperature until sectioning. Serial 4-5 μm sections were cut and adhered onto microscope slides. Paraffin was removed from the sections using Xylene; the samples were rehydrated, and processed using the streptavidin-biotin-peroxidase complex immunohistochemical technique. To ascertain immunoreactivity, antigen unmasking was performed by microwave treatment with 10 mM citrate buffer. Incubation with 10% normal goat serum in phosphate-buffered saline (PBS) was performed to eliminate nonspecific staining. After incubating for five minutes in 3% hydrogen peroxide, the slides were then incubated for 30 minutes at room temperature with primary antibody, VEGF-specific mouse monoclonal IgG (dilution 1:25; Dako). Detection of primary antibody was achieved with a secondary antibody detection kit (LSAB+kit, Dako, Denmark). Bound antigens were visualized using 3, 3-diaminobenzidine as a chromogen. Finally, the sections were counterstained with Mayer's hematoxylin, dehydrated, and mounted for analysis. Negative control was performed by incubating with Tris-buffered saline (TBS) instead of primary antibody. Colon carcinoma, shown to strongly express VEGF, was used as positive control.

### Immunohistochemical analysis

We intended to focus on the positivity in viable tumor tissue and to analyze the "hot spots" of immunoreactivity. The cells showing positive staining for VEGF were defined morphologically by hematoxylin and eosin (H&E) staining, using the serial sections. We compared immunohistochemical stains with preceding H&E slides to ascertain the exact location of immunoreactivity. Only cancer cells immunostained for VEGF were measured. The number of positive cells per 200 × field was assessed. In each slide three fields were evaluated. Semiquantitative expression levels of VEGF were determined by assessing both the percentage and intensity of stained tumour cells. The percentage of positive cells was rated as follows: cases with <1% positive cells were rated as 0 point, 1-25% positive cells were rated 1 point; 26-50% positive cells, 2 points; 51-75% positive cells, 3 points; 76-100% positive cells, 4 points. The staining intensity was rated as follows: 1 point, weak intensity; 2 points, moderate intensity; 3 points, strong intensity. Points for staining intensity and percentage of positive cells were added, and specimens were classified into 2 groups according to their overall score: weak expression 0-2 points; and strong expression, 3-7 points.

### Statistical analysis

Descriptive statistics and 95% confidence intervals were calculated to describe data. Data distribution was analyzed with the Smirnov-Kolmogorov test. According to the type of distribution, an appropriate parametric or an equivalent non-parametric test was used. The cutoff value for determining VEGF low and high expression score was performed by the receiver operating characteristic (ROC) curve analysis [28]. The relationship between VEGF expression data and gender, age, tumour stage, histology, stem cell transplantation therapy and survival was estimated by applying the Fisher's exact test [29]. Spearman's coefficient of rank correlation (rho) was determined to assess correlation between tumour stage and VEGF score, as well as VEGF score and survival. Overall survival (OS) was defined as the interval between the time of established diagnosis and patient's death. Univariate analysis of OS was performed as outlined by Kaplan and Meier [30]. Statistical significance of differences in survival between the patients groups with respect to gender, age, stage, histology, VEGF staining intensity and transplantation therapy was estimated using the log-rank test. Statistical analysis was performed using GrafPad Prism 5 (GrafPad Software, Inc, San Diego, CA) computer program. The Cox proportional hazards model was used for multivariate analysis to determine independent predictors of overall survival, and was carried out using MedCalc version 10.4 (MedCalc Software bvba, Mariakerke, Belgium) computer program [31]. Differences were considered significant at P < 0.05.

## Results

### Patient sample classifications

We examined tumour samples of 56 NB patients at disease onset. Patient characteristics are detailed in Table 1 and Table 2. The median patient follow-up time was 27 months (range, 1.0 to 180.0 months). The overall survival rate was 62,5%. Regarding age and gender at diagnosis, the mean age was 35,5 months (range 2 months to 12 years), 20 patients (35.7%) were ≤ 18 months of age, and 36 patients (64.3%) were >18 months old. 35 patients (62.5%) were males, and 21 patients (37.5%) females. Depending of the disease stage, we separated our patients into two groups: low stage (stage 1 and 2) and high stage (stage 3 and 4), as well as favourable and unfavourable histology according to the criteria reported by Shimada, *et al *[26,27]. Thirty-seven patients had high stage disease and eighteen had low stage disease. One patient had 4S stage disease. Twenty-three patients had favourable and thirty-thee patients had unfavourable histology. There was no statistically significant correlation between age (≤ 18 months/> 18 months) and disease stage (low/high) (P = 0.244), or between stage and histology (favourable/unfavourable) (P = 0.750) as determined by Fisher's exact test. Also no significant correlation between histology and age (≤ 18 months/> 18 months) was seen (P = 0.209).

**Table 2:** Patient characteristics

Patient no./sex/age (mo) at diagnosis	Tumour stage at diagnosis₤	Histologic subtype†	Age-linked classification‡	VEGF% points§	VEGF intensity¶	VEGF score^®^	Clinical outcome@
1/m/18	2	1a	U	1	2	3	S
2/m/36	2	4	U	1	1	2	S
3/m/18	2	1b	F	2	2	4	S
4/m/18	2	1a	U	2	2	4	S
5/f/37	2	2	F	1	1	2	S
6/m/10	3	1b	F	1	1	2	S
7/f/34	2	3	F	1	1	2	S
8/m/121	2	3	F	2	2	4	S
9/m/71	3	3	F	1	2	3	D*
10/m/3	1	1a	F	3	2	5	S
11/f/3	4s*	1a	U	1	1	2	S
12/f/33	2	1a	U	1	2	3	S
13/m/48	3	1a	U	4	3	7	S
14/m/53	3	2	F	1	2	3	S
15/m/18	3	1a	U	0	0	0	S
16/f/46	2	2	F	2	3	5	S
17/f/2	2	1b	F	4	3	7	S
18/m/78	2	2	F	1	1	2	S
19/m/4	3	1b	F	2	3	5	S
20/m/2	2	1a	F	1	2	3	S
21/m/2	2	1a	U	0	0	0	S
22/f/18	3	1b	F	1	2	3	S
23/f/14	2	1b	F	1	1	2	S
24/m/72	3	2	F	3	3	6	S
25/m/144	4	1a	U	2	3	5	D
26/f/24	4	1a	U	2	3	5	D
27/m/120	4	1a	U	2	3	5	D
28/m/38	3	2	F	2	2	4	D
29/m/120	4	1a	U	1	2	3	D
30/m/35	4	1a	U	1	2	3	D
31/f/61	4	1a	U	2	2	4	D
32/m/97	2	1a	U	1	2	3	D
33/m/84	3	1b	U	3	2	5	D
34/f/23	4	1b	U	2	2	4	D
35/f/42	4	1a	U	4	3	7	D
36/f/5	4	1a	U	2	3	5	D
37/m/36	3	1a	U	3	2	5	D
38/f/48	4	1a	U	4	3	7	D
39/m/10	4	1a	U	1	2	3	D
40/f/122	2	1a	U	1	1	2	S
41/m/38	4	1a	U	3	3	6	S
42/f/35	3	2	F	1	4	6	S
43/m/30	1	2	U	1	1	2	S
44/m/3	4	1b	F	2	2	4	S
45/f/12	4	1b	F	2	3	5	S
46/f/17	3	1a	U	2	3	5	S
47/m/18	4	1a	U	2	3	5	S
48/m/36	3	2	F	1	1	2	S
49/m/66	4	1b	U	1	2	3	S
50/m/12	4	1a	F	2	3	5	S
51/m/36	3	2	F	3	2	5	S
52/f/35	4	4	U	1	3	4	D
53/f/54	4	1a	U	1	2	3	D
54/m/54	4	4	U	2	3	5	D
55/m/60	3	1a	U	3	1	4	D
56/f/56	4	1b	U	1	2	3	D

₤ Tumour staging at diagnosis according to INSS^25^;

† Shimada histopathologic classification^26 ^1a: Stroma poor, undifferentiated; 1b: Stroma poor, differentiating; 2: Stroma rich, intermixed; 3: Stroma rich, well differentiated; 4: Stroma rich, focal nodular

‡ Prognostic groups according to the age-linked classification of Shimada^26 ^F: favourable; U: unfavourable;

§VEGF % (percentage of positive VEGF cells) 0 point: <1% positive cells, 1 point: 1-25% positive cells; 2 points: 26-50% positive cells; 3 points: 51-75% positive cells; 4 points: 76-100% positive cells

¶VEGF intensity (staining intensity) 1: weak intensity; 2: moderate intensity; 3: strong intensity

^® ^VEGF score: points for staining intensity and percentage of positive cells were added

@ S: surviving, D: dead, D*: one patient died in relapse of tumour 5,5 yr after diagnosis

4S* in a infant with small primary tumour and metastatic disease involving the skin, liver, limited infiltration of the bone marrow

### VEGF is expressed in NB samples

**Figure 1:**
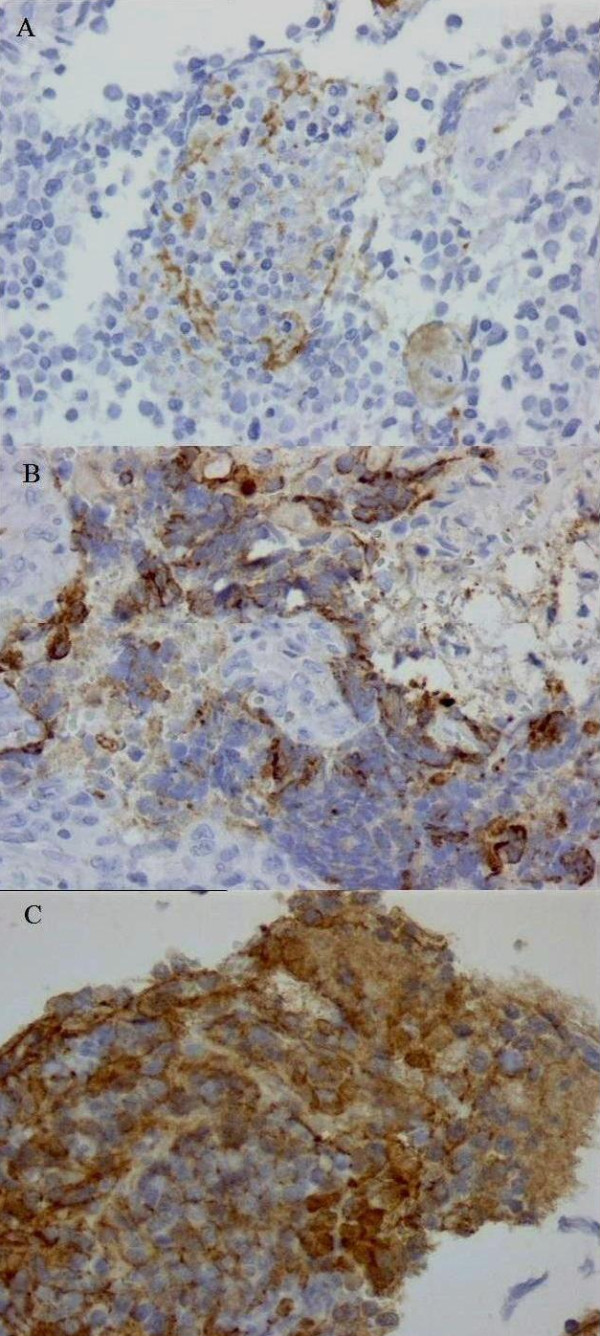
**Immunohistochemical staining for VEGF in different NB pathohistological sections**. Low VEGF expression score with low grade intensity and 1-25% tumour cell positivity **(A)**; High VEGF expression score with high grade intensity and 25-50% tumour cell positivity **(B)**; High VEGF expression score with moderate grade intensity and 75%-100% tumour cell positivity **(C)**. Objective = 40×.

According to the criteria proposed by Volms, *et al*., brown granules in the cytoplasm of tumour cells were identified to be VEGF positive [32]. VEGF staining was found in 54 of 56 tumours (96.4%), and only 2 tumours (3.6%) had negative VEGF immunoreactivity. Most of the specimens (76, 7%) had 1-50% positive tumour cells, and 76.8% of specimens had moderate to strong staining intensity. By assessing both the percentage and intensity of stained tumour cells, specimens were classified into 2 groups according to their overall score. By ROC curve analysis, and cut off value of >2.5 (sensitivity 100%, specificity 56.25%), tumours were distinguished with high (3-7) and low (0-2) VEGF expression scores. Low VEGF expression scores were found in 12 NB (21.4%) and high expression score in 44 NB (78.6%) (Table 3). Figure 1 is showing VEGF immunohistochemical staining in different NB pathohistological sections.

**Table 3:** Immunoreactivity of VEGF, and the number of patients

Category	Number of patients (%)	alive/dead
Percentage of positive		
tumour cells (P)		
<1%	2 (3.6%)	2/0
1-25%	25 (44.6%)	17/8
26-50%	18 (32.1%)	10/8
51-75%	7 (12.5%)	4/3
76-100%	4 (7.1%)	2/2
		
Staining intensity (I)		
Negative	2 (3.6%)	2/0
Weak	11 (19.6%)	10/1
Moderate	24 (42.9%)	12/12
Strong	19 (33.9%)	11/8
		
Expression score (P+I)		
Low (0-2)	12 (21.4%)	12/0
High (3-7)	44 (78.6%)	23/21

### Correlation of VEGF expression with clinicopathological characteristics and survival

VEGF expression and clinicopathological characteristics are detailed in Table 4. Fisher's exact test was performed. We did not observe significant correlation between VEGF expression (high/low) and gender (P = 0.7477), age >18 months/≤ 18 months old (P = 0.2701), or histology (favourable/unfavourable) (P = 0.27). Also, there was no significant difference in VEGF expression between the transplant and non-transplant patients (P = 0.7378).

**Table 4:** VEGF expression and other clinicopathologic factors

Characteristics	VEGF score	
	Low	High
	No. patients	
Total number	12	44
		
Gender		
Male	7	28
Female	5	16
		
Age		
>18 months old	4	32
≤ 18 months old	8	12
		
Histologic subtype		
Stroma-rich		
Well differentiated	1	2
Intermixed	3	7
Focal nodular	1	2
Stroma-poor		
Undifferentiated	6	24
Differentiating	1	9
		
Histology		
Favourable	5	18
Unfavourable	7	26
		
Stage		
1	1	2
2	7	8
3	3	17
4	0	17
4s	1	0
		
Transplant		
No	9	30
Yes	3	14
		
Survival		
Alive	12	23
Dead	0	21

There was significant association between advanced disease stage and high VEGF expression as determined by Fisher exact test (P = 0.0014), and significant correlation between high VEGF expression score and high tumour stage as determined by Spearman's coefficient of rank, (rho = 0.453, P = 0.0005).

The VEGF expression score was significantly higher in the group of non-survival patients compared to the group of patients that survived more than 5 years, as determined by Mann Whitney test (P < 0.0001). Also, significant correlation between VEGF expression and survival was determined by Spearman's coefficient of rank (rho = -0.472, P = 0.0002). All patients with low VEGF expression score survived.

**Figure 2:**
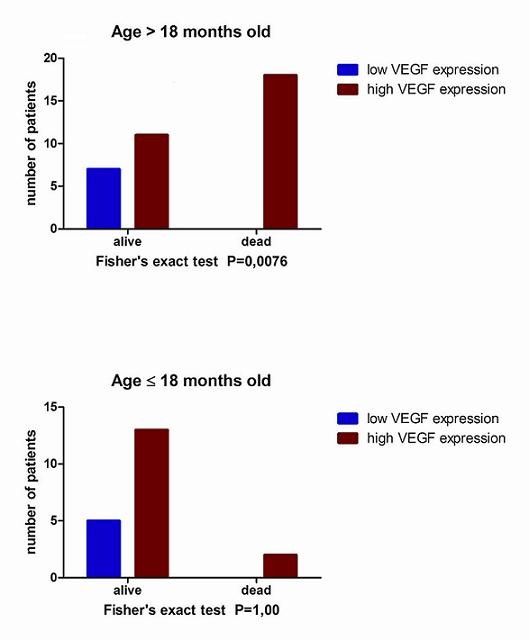
**The impact of VEGF on survival in different age groups**. Expression of VEGF has impact on survival in the patients > 18 months old **(A)**. VEGF expression is not statistically significant for survival in the group of patients ≤ 18 months old **(B)**.

Interestingly, in the group of patients ≤ 18 months old we did not observe any correlation between VEGF expression and tumour stage (Spearman's coefficient of rank rho = 0.17, P = 0.46), opposite to the patients > 18 months old (rho = 0.635, P < 0.0001). In the same group of patients (≤ 18 months old), we also did not observe any correlation between VEGF expression score and survival (Spearman's coefficient of rank rho = 0.19, P = 0.42; Fisher's exact test P = 1.0), contrary to the group of patients > 18 months old (Spearman's coefficient of rank rho = 0.49, P = 0.0023; Fisher's exact test P = 0.0076), (Figure 2).

### Univariate survival analysis

**Figure 3:**
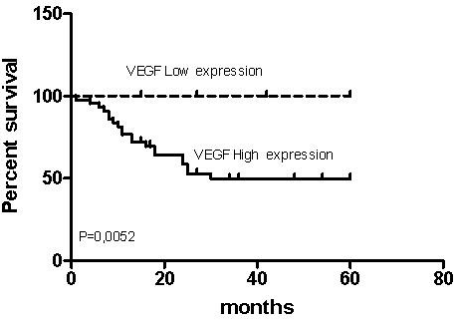
**VEGF and survival by Kaplan-Meier analysis**. Expression of VEGF is a significant prognostic factor. Kaplan-Meier analysis of overall survival for all NB patients according to high and low VEGF expression (P = 0.0052).

Log-rank test was performed. There were significant differences in survival rates in the groups of patients with ≤ and > 18 months old (P = 0.0069; Table 5). Patients > 18 months old had lower survival rate than patients ≤ 18 months old. Patients with advanced stage tumours (Stage 3, 4), had lower survival rate when compared to patients with low stage tumours (P = 0.0006; Table 5). There were significant differences in survival rates in the groups of patients with favourable and unfavourable histology (P < 0.0001; Table 5). Patients with high VEGF expression had short median OS (30 months). Survival curve of the VEGF low expression group was significantly higher, and OS longer, compared to the VEGF high expression group (P = 0.0053; Figure 3, Table 5). Survival was not correlated with sex (P = 0.45; Table 5).

**Table 5:** Overall survival rates and univariate analysis of patients with NB according to clinicopathologic factors

Variable	Number of patients	Overall survival rates	Log-rank Test
Gender			
boys	35	68.6%	P = 0.4497
girls	21	57.1%	
			
Age			
≤ 18 months	20	90%	P = 0.0069
> 18 months	36	50%	
			
Stage			
high	37	50.0%	P = 0.0006
low	18	94.4%	
			
Histology			
favourable	23	95.7%	P < 0.0001
unfavourable	33	42.4%	
			
VEGF expression			
high	44	54.5%	P = 0.0053
low	12	100.0%	
			
Risk group			
high*	34	44.1%	P < 0.0001
low**	22	95.5%	

Abbreviations:*high VEGF expression (score3-7) together with high disease stage (Stage III, IV);

**all others

### High risk patients

**Figure 4:**
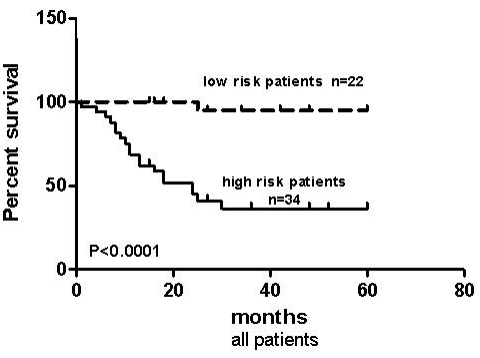
**High risk group and survival by Kaplan-Meier analysis**. High risk group has short overall survival (OS) (24.00 months). Kaplan-Meier analysis of OS according to risk groups* for NB patients (P < 0.0001). Abbreviation: risk groups* = high risk group: patients with high disease stage (stage III, IV) and high VEGF expression score (3-7); low risk group: all other patients.

Patients with high disease stage (Stage 3, 4) and high VEGF expression score (score 3-7) had short median OS (24 months). These patients had significantly lower survival rate than all other patients (p < 0.0001; Table 5, Figure 4). The non-transplant patients with high stage disease and high VEGF expression score (high risk patients), had the shortest median OS (13 months) and significantly lower survival rate when compared to all other (low risk) non transplant patients (p < 0.0001). Among the high-risk patients (high stage and high VEGF expression), those patients who had bone marrow transplants had significantly better survival rate (undefined median OS) when compared to non-transplant patients (median OS 13 months) (p = 0.0237).

### Tumour stage and VEGF expression, as one combined variable - the significant mortality predictor by multivariate analysis

The full Cox proportional-hazards regression model containing all predictors was statistically significant (P < 0.001), indicating that this model was able to distinguish between survival and non-survival. As shown in Table 6, three predictor variables significantly affected the model, unfavourable histology, high disease stage, and transplantation. Although we did not demonstrate the role of VEGF as an independent prognostic factor by multivariate analysis, the combination of high tumour stage and high VEGF expression as one complex predictor variable, became the strongest mortality predictor by Cox proportional-hazards regression model (OR = 26.1695, 95% CI = 2.9741 to 230.2670, P = 0.0034; Table 7). These results showed that prognostic prediction might be improved by taking into account both VEGF expression and disease stage.

**Table 6:** Cox proportional-hazards regression model* for NB patients overall survival

Covariate	P	OR**	95% CI***of OR
High stage	0.0238	11.3891	1.3949 to 92.9926
VEGF expression score	0.3831	1.1790	0.8159 to 1.7038
Unfavourable histology	0.0073	16.4610	2.1432 to 126.4302
Age older than 18 months	0.1988	3.0418	0.5624 to 16.4532
Without transplantation	0.0295	3.2280	1.1298 to 9.2227

*Overall model fit χ^2 ^= 42.105 P < 0.0001

Abbreviations: **Odds ratio; *** Confidence interval

**Table 7:** Cox proportional-hazards regression model* including High risk** covariate for NB patients overall survival

Covariate	P	OR***	95%CI****of OR
High risk	0.0034	26.1695	2.9741 to 230.2670
Without transplantation	0.0111	4.2160	1.3949 to 12.7425
Unfavourable histology	0.0052	20.4384	2.4824 to 168.2770
Age older than 18 months 0.6819		1.4019	0.2809 to 6.9955

*Overall model fit χ^2 ^= 45.904 P < 0.0001

Abbreviations: ** High VEGF expression (score3-7) together with high disease stage (Stage III, IV);***Odds ratio; ****Confidence interval

## Discussion

So far, in some adult solid tumours semi-quantitative VEGF expression has been successfully evaluated by immunohistochemistry, and VEGF has been reported to be an independent prognostic factor [11-15]. We performed similar investigation in the cohort of patients with neuroblastoma which is the most frequent extra cranial solid malignancy in children and has a great mortality rate. In order to evaluate the prognostic significance of VEGF expression in NB patients, and estimate its diagnostic usefulness in a routine clinical practice, we have attempted to establish semi-quantitative VEGF score. As we intended to focus on positivity in viable tumour tissue, the most reliable method was immunohistochemistry. To our knowledge, this is the first time that VEGF immunohistochemistry score has been evaluated in NB patients. We analyzed "hot spots" of immunoreactivity which could be easily missed by other techniques.

In our cohort VEGF positive immunostaining was found in 96.4% of all NB tumour specimens tested, with most samples having moderate to strong staining intensity (78.6%). Despite some differences in scoring systems described in different studies, the frequency of VEGF positive tumours in this study was higher than in adult cancers [11,13-15]. It can be explained by NB-specific biology and significant tumour tissue hypoxia [8,33,34].

No correlation between VEGF expression and gender, age, or histology was found. However, there was significant correlation between high stage and high VEGF expression, and between high VEGF expression and short survival. Contrary to the patients with high VEGF expression, all patients with low VEGF expression survived.

These results support the hypothesis of a dual function for VEGF in autocrine tumour growth. In addition to its effects on angiogenesis, VEGF may affect NB cell growth, directly, and could be an autocrine growth factor [35]. In addition to stimulating angiogenesis in tumour growth, VEGF also mediates neuroprotection promoting neuroblastoma cellular survival by increasing Bcl-2 and pro-caspase 3 expressions [36].

Additional trials also confirm the correlation between VEGF expression and the grade of NB [5,35,37,38]. VEGF levels in the sera of metastatic NB patients and other paediatric solid tumour patients are much higher than in non-metastatic patients [39]. Other authors did not find correlation between VEGF expression and disease stage, but they found association between high VEGF expression and unfavourable histology [19]. Perhaps, the differences between the results were caused by small patient groups and different methods of VEGF evaluation. Larger multicentric studies are needed to obtain more reproducible results. Also, new experimental models to study the angiogenic and invasive potential of NB tumours cells are still needed in order to further investigate human tumour progression and anti-angiogenic molecule screening [40,41].

As we mentioned, we found significant correlation between high stage and high VEGF expression, and strong correlation between high VEGF expression and short survival in the cohort of our NB patient, except in the patients with age ≤ 18 months old. Patients younger than 18 months have a good prognosis, and spontaneous tumour maturation/regression can happen due to favourable autocrine and paracrine interactions among tumour cells. We suppose that in these tumours the effects of VEGF could be diminished by stimulators of tumour maturation, but further prospective designed neuroblastoma angiogenesis/anti-angiogenesis studies are needed to draw conclusions. Maybe one of these factors is Pigment epithelium-derived factor (PEDF) which is inhibitor of angiogenesis and inducer of neural differentiation [42]. In the study of Crawford SE, *et al*. (2001), **"**undifferentiated neuroblastoma tumour cell secretions were angiogenic primarily due to vascular endothelial growth factor, and secretions of Schwann cells were anti-angiogenic due to PEDF. In addition, PEDF was the major factor responsible for Schwann cell's ability to induce tumour cell differentiation in vitro and recombinant PEDF had the same effect in vitro and in vivo. Thus PEDF may serve as a multifunctional antitumor agent in neuroblastomas" [42].

Survival rates of our NB patients were analyzed according to gender, age, stage, histology, and VEGF expression. In accordance with previous reports (1), age > 18 months was a significant prognostic factor. By univariate analysis, tumour stage, favourable/unfavourable histology and VEGF immunoreactivity were also found to be significant prognostic factors for overall survival. By combining VEGF expression and disease stage the prognostic value for survival was even more improved. Patients with high tumour stage and high VEGF expression were high-risk, with short median of overall survival (OS) (24 months). Among this group, there were significant differences in OS between transplant (undefined median OS), and non-transplant patients (13 months median OS). Multimodal therapy with hematopoietic stem cell transplantation significantly improved survival of these high risk patients. Perhaps survival rates could be further improved by adding bevacizumab in their therapy because in addition to its antiangiogenic and proapoptotic properties, bevacizumab can transiently "normalize" the abnormal structure and function of tumour vasculature to make it more efficient for oxygen and drug delivery [43]. If bevacizumab treatment suppresses NB progression in the setting of minimal residual disease, it would likely be a good therapy option post stem cell transplantation for high VEGF expression, high risk patients [44].

In multivariate analysis by the Cox regression model, Shimada histopathology age-linked classification, tumour stage and hematopoietic stem cell transplantation had significance as independent prognostic factors for overall survival. Although we did not demonstrate the role of VEGF expression score as an independent prognostic factor by multivariate analysis, the combination of high tumour stage and high VEGF expression as one complex predictor variable was the strongest mortality predictor by Cox proportional-hazards regression model.

As tumour angiogenesis correlates with metastatic disease, N-*myc *amplification, and poor outcome in human neuroblastoma, and some studies suggest that N-*myc *may function in part by promoting angiogenesis via VEGF, it would be important to compare N-*myc *amplification with VEGF expression in the clinical trials [3,41]. Due to our failure to obtain DNA of sufficient quality when we tried to prepare paraffin-embedded material for molecular biology study, we were not able to correlate N-*myc *amplification level and VEGF expression. Nevertheless, our results indicated that VEGF expression should be considered in the diagnostic workup of children with neuroblastoma, especially in those older than 18 months and with advanced disease stage.

## Conclusion

This study suggests that VEGF, a critical regulator of tumour angiogenesis, might serve as an important neuroblastoma prognostic biological marker in a routine clinical practice. It can be used to identify neuroblastoma high risk patients in combination with tumour stage and other relevant risk factors. Furthermore, VEGF expression would be useful in determining the necessity for stem cell transplantation, determining follow-up strategies and anti-angiogenic therapy trials.

## Competing interests

The authors declare that they have no competing interests.

## Authors' contributions

GJ made conception, designed and coordinated the study, collected samples, analyzed data, carried out data interpretation, and drafted the manuscript. SS participated in the conception and design of the study, performed the revaluation and new grading of the histological samples, carried out the immunohistological analysis, and participated in drafting of manuscript. SČ participated in the conception and design of study, and in drafting of manuscript. JS and AB helped to collect the samples and to draft the manuscript. All authors read and approved the final manuscript.

## References

[B1] Maris JM Hogarty MD Bagatell R Cohn SL Neuroblastoma Lancet 2007 369 2106 2120 10.1016/S0140-6736(07)60983-0 17586306

[B2] Hanahan D Folkman J Patterns and emerging mechanisms of the angiogenic switch during tumorigenesis Cell 1996 86 353 364 10.1016/S0092-8674(00)80108-7 8756718

[B3] Meitar D Crawford SE Rademaker AW Cohn SL Tumor angiogenesis correlates with metastatic disease, N-*Myc *amplification, and poor outcome in human neuroblastoma J Clin Oncol 1996 14 405 414 8636750 10.1200/JCO.1996.14.2.405

[B4] Ribatti D Vacca A Nico B De Falco G Giuseppe Montaldo P Ponzoni M Angiogenesis and anti-angiogenesis in neuroblastoma Eur J Cancer 2002 38 750 757 10.1016/S0959-8049(01)00337-9 11937307

[B5] Eggert A Ikegaki N Kwiatkowski J Zhao H Brodeur GM Himelstein BP High-Level Expression of Angiogenic Factors is Associated with Advanced Tumor Stage in Human Neuroblastoma Clin Cancer Res 2000 6 1900 1908 10815914

[B6] Chlenski A Liu S Crawford SE Volpert OV DeVries GH Evangelista A Yang Q Salwen HR Farrer R Bray J Coh SL SPARC is a key Schwannian-derived inhibitor controlling neuroblastoma tumor angiogenesis Cancer Res 2002 62 7357 7363 12499280

[B7] Goldberg MA Schneider TJ Similarities between the oxygen sensing mechanisms regulating the expression of vascular endothelial growth factor and erythropoietin J Biol Chem 1994 269 4355 4359 8308005

[B8] Rössler J Taylor M Geoerger B Lagodny JF Peschka-Süss R Niemeyer C Vassal G Angiogenesis as a target in neuroblastoma Eur J Cancer 2008 44 1645 1656 10.1016/j.ejca.2008.05.015 18614349

[B9] Drozynska E Izycka-Swieszewska E Balcerska A Bodalski J Bohosiewicz J Brozyna A Bubała H Chybicka A Grajkowska W Koltan S Madziara W Rybczyńska A Słociak M Sońta-Jakimczyk D Stolarska M Perek D Wachowiak J Wysocki M Analysis of microvascular density and the expression of vascular endothelial growth factor (VEGF) and its membrane receptor Flk-1 in neuroblastoma Med Wieku Rozwoj 2006 10 745 755 17317905

[B10] Ghanem MA van Steenbrugge GJ Sudaryo MK Mathoera RB Nijman JM Kwas ThH van der Expression and prognostic relevance of vascular endothelial growth factor (VEGF) and its receptor (FLT-1) in nephroblastoma JCP 2003 56 107 113 12560388 10.1136/jcp.56.2.107 PMC1769871

[B11] Kulke MH Odze RD Mueller JD Wang H Redston M Bertagnolli MM Prognostic significance of vascular endothelial growth factor and cyclooxygenase 2 expression in patients receiving preoperative chemoradiation for esophageal cancer J Thorac Cardiovasc Surg 2004 127 1579 1586 10.1016/j.jtcvs.2003.12.034 15173710

[B12] Zafirellis K Agrogiannis G Zachaki A Gravani K Karameris A Kombouras C Prognostic Significance of VEGF Expression Evaluated by Quantitative Immunohistochemical Analysis in Colorectal Cancer J Surg Res 2008 147 99 107 10.1016/j.jss.2007.05.041 17655863

[B13] Aoyagi K Kouhuji K Yano S Miyagi M Imaizumi T Takeda J Shirouzu K VEGF significance in peritoneal recurrence from gastric cancer Gastric Cancer 2005 8 155 163 10.1007/s10120-005-0329-4 16086118

[B14] Yilmaz A Ernam D Unsal E Demirag F Atikcan Ş Taştepe I Vascular Endothelial Growth Factor Immunostaining Correlates with Postoperative Relapse and Survival in Non-Small Cell Lung Cancer Arch Med Res 2007 38 764 768 10.1016/j.arcmed.2007.04.005 17845896

[B15] Du JR Jiang Y Zhang YM Fu H Vascular endothelial growth factor and microvascular density in esophageal and gastric carcinoma World J Gastroenterol 2003 9 1604 1606 12854174 10.3748/wjg.v9.i7.1604 PMC4615515

[B16] Yang JC Haworth L Sherry RM Hwu P Schwartzentruber DJ Topalian SL Steinberg SM Chen HX Rosenberg SA A randomized trial of bevacizumab, an anti-vascular endothelial growth factor antibody, for metastatic renal cancer N Engl J Med 2003 349 427 434 12890841 10.1056/NEJMoa021491 PMC2275324

[B17] Hurwitz H Fehrenbacher L Novotny W Cartwright T Hainsworth J Heim W Berlin J Baron A Griffing S Holmgren E Ferrara N Fyfe G Rogers B Ross R Kabbinavanr F Bevacizumab plus irinotecan, fluorouracil, and leucovorin for metastatic colorectal cancer N Engl J Med 2004 350 2335 2342 10.1056/NEJMoa032691 15175435

[B18] Herbst RS Johnson DH Mininberg E Carbone DP Henderson T Kim ES Blumenschein G Jr Lee JJ Liu DD Truong MT Hong WK Tran H Tsao A Xie D Ramies DA Mass R Seshagiri S Eberhard DA Kelley SK Sandler A Phase I/II trial evaluating the anti-vascular endothelial growth factor monoclonal antibody bevacizumab in combination with the HER-1/epidermal growth factor receptor tyrosine kinase inhibitor erlotinib for patients with recurrent non-small-cell lung cancer J Clin Oncol 2005 23 2544 2555 10.1200/JCO.2005.02.477 15753462

[B19] Fukuzawa M Sugiura H Koshinaga Tatsuo S Expression of Vascular Endothelial Growth Factor and Its Receptor Flk-1 in Human Neuroblastoma Using In Situ Hybridization J Pediatr Surg 2002 37 1747 1750 10.1053/jpsu.2002.36712 12483647

[B20] Rössler J Breit S Havers W Schweigerer L Vascular endothelial growth factor expression in human neuroblastoma: up-regulation by hypoxia Int J Cancer 1999 81 113 117 10.1002/(SICI)1097-0215(19990331)81:1<113::AID-IJC19>3.0.CO;2-L 10077161

[B21] Ootsuka S Asami S Sasaki T Yoshida Y Nemoto N Shichino H Chin M Hideo Mugishima H Suzuki T Analyses of Novel Prognostic Factors in Neuroblastoma Patients Biol Pharm Bull 2007 30 2294 2299 10.1248/bpb.30.2294 18057715

[B22] Ribatti D Marimpietri D Pastorino F Brignole C Nico B Vacca A Ponzoni M Angiogenesis in Neuroblastoma Ann NY Acad Sci 2006 1028 133 142 10.1196/annals.1322.014 15650239

[B23] Shusterman S Maris JM Prospects for therapeutic inhibition of neuroblastoma angiogenesis Cancer Lett 2005 228 171 179 10.1016/j.canlet.2005.01.049 15927358

[B24] Glade Bender JL Adamson PC Reid JM Xu L Baruchel S Shaked Y Kerbel RS Cooney-Qualter EM Stempak D Chen HX Nelson MD Krailo MD Ingle AM Blaney SM Kandel JJ Yamashiro DJ Phase I Trial and Pharmacokinetic Study of Bevacizumab in Pediatric Patients With Refractory Solid Tumors J Clin Oncol 2008 26 399 405 10.1200/JCO.2007.11.9230 18202416

[B25] Brodeur GM Pritchard J Berthold F Carlsen NL Castel V Castelberry RP De Bernardi B Evans AE Favrot M Hedborg F Revisions of the international criteria for neuroblastoma diagnosis, staging, and response to treatment J Clin Oncol 1993 11 1466 1477 8336186 10.1200/JCO.1993.11.8.1466

[B26] Shimada H Ambros IM Dehner LP Hata J Joshi VV Roald B Stram DO Gerbing RB Lukens JN Matthay KK Robert P Castleberry RP The International Neuroblastoma Pathology Classification (the Shimada System) Cancer 1999 86 364 372 10.1002/(SICI)1097-0142(19990715)86:2<364::AID-CNCR21>3.0.CO;2-7 10421273

[B27] Shimada H Chatten J Newton WA Jr Sachs N Hamoudi AB Chiba T Marsden HB Misuqi K Histopathologic prognostic factors in neuroblastic tumors: definition of subtypes of ganglioneuroblastoma and an age-linked classification of neuroblastomas J Natl Cancer Inst 1984 73 405 416 6589432 10.1093/jnci/73.2.405

[B28] Søreide K Receiver-operating characteristic curve analysis in diagnostic, prognostic and predictive biomarker research JCP 2009 62 1 5 18818262 10.1136/jcp.2008.061010

[B29] Fleiss J Levin B Cho Paik M Statistical Methods for Rates and Proportions 1973 New York: John Wiley & Sons, Inc

[B30] Kaplan EL Meier P Nonparametric estimation from incomplete observations J Am Stat Assoc 1958 53 457 481 10.2307/2281868

[B31] Therneau TM Grambsch PM Modeling Survival Data: Extending the Cox Model 2000 New York: Springer

[B32] Volm M Koomägi R Mattern J Prognostic value of vascular endothelial growth factor and its receptor Flt-1 in squamous cell lung cancer Int J Cancer 1997 74 64 68 10.1002/(SICI)1097-0215(19970220)74:1<64::AID-IJC11>3.0.CO;2-I 9036871

[B33] Rössler J Stolze I Frede S Freitag P Schweigerer Havers W Fandrey J Hypoxia- induced erythropoietin expression in human neuroblastoma requires a methylation free HIF-1 binding site J Cell Biochem 2004 93 153 161 10.1002/jcb.20133 15352172

[B34] Stolze I Berchner-Pfannschmidt U Freitag P Wotzlaw C Rössler J Frede S Acker H Fandrey J Hypoxia-inducible erythropoietin gene expression in human neuroblastoma cells Blood 2002 100 2623 2628 10.1182/blood-2001-12-0169 12239177

[B35] Langer I Vertongen P Perret J Fontaine J Atassi G Robberecht P Expression of Vascular Endothelial Growth Factor (VEGF) and VEGF Receptors in Human Neuroblastomas Med Pediatr Oncol 2000 34 386 393 10.1002/(SICI)1096-911X(200006)34:6<386::AID-MPO2>3.0.CO;2-3 10842244

[B36] Wang D Weng Q Zhang L He Q Yang B VEGF and Bcl-2 Interact Via MAPKs Signaling Pathway in the Response to Hypoxia in Neuroblastoma Cell Mol Neurobiol 2009 29 391 401 10.1007/s10571-008-9331-9 19048366 PMC11506184

[B37] Marcus K Johnson M Adam RM O'Reilly MS Donovan M Atala A Freeman MR Soker S Tumor cell-associated neuropilin-1 and vascular endothelial growth factor expression as determinants of tumor growth in neuroblastoma Neuropathology 2005 25 178 187 10.1111/j.1440-1789.2005.00610.x 16193833

[B38] Nowicki M Konwerska A Ostalska-Nowicka D Katarzyna Derwich K Miskowiak B Kondraciuk B Samulak D Witt M Vascular endothelial growth factor (VEGF)-C - a potent risk factor in children diagnosed with stadium 4 neuroblastoma Folia Histochem Cytobiol 2008 46 493 499 10.2478/v10042-008-0067-7 19141404

[B39] El-Houseini ME Abdel-Azim SA El-Desouky GI Abdel-Hady S El-Hamad MF Kamel AM Clinical Significance of Vascular Endothelial Growth Factor (VEGF) in Sera of Patients with Pediatric Malignancies J Egypt Natl Canc Inst 2004 16 57 61 15716999

[B40] Mangieri D Nico B Coluccia A Vacca Ponzoni M Ribatti D An alternative in vivo system for testing angiogenic potential of human neuroblastoma cells Cancer Lett 2009 277 199 204 10.1016/j.canlet.2008.12.014 19150583

[B41] Zaghloul N Hernandez SL Bae JO Huang J Fisher JC Lee A Kadenhe-Chiweshe A Kandel JJ Yamashiro DJ Vascular endothelial growth factor blockade rapidly elicits alternative proangiogenic pathways in neuroblastoma Int J Oncol 2009 34 401 407 19148474 PMC3070359

[B42] Crawford SE Stellmach V Ranalli M Huang X Huang L Volpert O De Vries GH Abramson LP Bouck N Pigment epithelium-derived factor (PEDF) in neuroblastoma: a multifunctional mediator of Schwann cell antitumor activity J Cell Sci 2001 114 4421 4428 11792807 10.1242/jcs.114.24.4421

[B43] Dickson PV Hamner JB Sims TL Fraga CH Ng CYC Rajasekeran S Hagedorn NL McCarville MB Stewart CF Davidoff AM Bevacizumab-Induced Transient Remodeling of the Vasculature in Neuroblastoma Xenografts Results in Improved Delivery and Efficacy of Systemically Administered Chemotherapy Clin Cancer Res 2007 13 3942 3950 10.1158/1078-0432.CCR-07-0278 17606728

[B44] Sims TL Williams RF Ng CY Rosati SF Spence Y Davidoff AM Bevacizumab suppresses neuroblastoma progression in the setting of minimal disease Surgery 2008 144 269 275 18656635 10.1016/j.surg.2008.04.009 PMC2525799

